# Nurses Advancing Geriatric Excellence through Simulation (NU‐AGE‐SIM) from Virtual Reality to Holograms

**DOI:** 10.1002/alz70858_103786

**Published:** 2025-12-26

**Authors:** Nicole P Blodgett, Duke University NU‐AGE‐SIM Team

**Affiliations:** ^1^ Duke Univeristy School of Nursing, Durham, NC, USA

## Abstract

Critical gaps in nursing education and workforce readiness exist to care for medically underserved older adults with cognitive decline. The **purpose** of this project is to describe the innovative use of an educational bundle (including virtual reality and holograms) to improve the care of older adults with cognitive decline.

**Methods:**

Nurses Advancing Geriatric Excellence through Simulation (NU‐AGE‐SIM) included a scaffolded approach: 1) didactic sessions about older adults with cognitive decline, assessment, and interventions anchored by Age Friendly Health Systems 4Ms (mentation, medication, mobility, and what matters). 2) Virtual reality to embody an older adult with cognitive decline and experience common issues in older adults (ex. hearing loss, vision changes, social isolation). 4) Life‐size holograms scenarios, co‐designed with clinical partners, allowed trainees to develop competency working with diverse, older adults with cognitive decline. 5) A clinical experience working directly with community dwelling older adults. Kolb's concrete experiences, active experimentation, and best practices in simulation were utilized.

**Results:**

Trainees (*n* = 20) reported they met learning objectives: demonstrate communication with member of healthcare team (97%); demonstrate empathy with older adult and caregiver (100%); and explore feelings about older adult challenges (98%). Reported competency in: assessment (95%), SPICES (100%), medications (79%), mentation (100%), mobility, (95%), and what matters (100%). NU‐AGE‐SIM trainees recommended virtual reality and holograms higher than clinical experiences and didactic. In **conclusion**, NU‐AGE‐SIM represents a vital step toward preparing a diverse nursing workforce capable of addressing the unique needs of older adults with cognitive decline.

